# Age-related differences in foot mobility in individuals with patellofemoral pain

**DOI:** 10.1186/s13047-018-0249-2

**Published:** 2018-02-15

**Authors:** Jade M. Tan, Kay M. Crossley, Bill Vicenzino, Hylton B. Menz, Shannon E. Munteanu, Natalie J. Collins

**Affiliations:** 1 Discipline of Podiatry, School of Allied Health, College of Science, Health and Engineering, La Trobe University, Melbourne, VIC 3086 Australia; 2 La Trobe Sport and Exercise Medicine Research Centre, La Trobe University, Melbourne, VIC 3086 Australia; 3 School of Health and Rehabilitation Sciences, The University of Queensland, St Lucia, Brisbane, QLD 4072 Australia; 4 Department of Mechanical Engineering, Melbourne School of Engineering, The University of Melbourne, Parkville, VIC 3010 Australia

**Keywords:** Patellofemoral pain, Foot mobility, Ageing

## Abstract

**Background:**

Age-related changes in midfoot mobility have the potential to influence success with foot orthoses intervention in people with patellofemoral pain (PFP). The aim of this study was to determine whether older people with PFP demonstrate less foot mobility than younger adults with PFP.

**Methods:**

One hundred ninety four participants (113 (58%) women, age 32 ± 7 years, BMI 25 ± 4.9 kg/m^2^) with PFP (≥ 6 weeks duration) were included, with foot mobility quantified using reliable and valid methods. K-means cluster analysis classified participants into three homogenous groups based on age. After cluster formation, univariate analyses of co-variance (covariates: sex, weight) were used to compare midfoot height mobility, midfoot width mobility, and foot mobility magnitude between age groups (significance level 0.05).

**Results:**

Cluster analysis revealed three distinct age groups: 18–29 years (*n* = 70); 30–39 years (*n* = 101); and 40–50 years (*n* = 23). There was a significant main effect for age for midfoot height mobility (*p* < 0.001) and foot mobility magnitude (*p* = 0.006). Post-hoc analyses revealed that midfoot height mobility differed across all three groups (moderate to large effect sizes), and that foot mobility magnitude was significantly less in those aged 40–50 years compared to those aged 18–25 years (moderate effect size). There were no significant main effects for age for midfoot width mobility (*p* > 0.05).

**Conclusion:**

Individuals with PFP aged 40–50 years have less foot mobility than younger adults with PFP. These findings may have implications for evaluation and treatment of older individuals with PFP.

## Background

Patellofemoral pain (PFP) is a common condition that can affect individuals of all ages, from adolescence to later life [1]. Characterised by anterior or retro-patellar knee pain, those affected typically experience symptoms during weight-bearing activities that load the patellofemoral (PF) joint, such as squatting, walking up and down stairs, and running [2].

Across the lifespan, foot orthoses are one physical modality used to manage symptoms of PFP. In younger adults with PFP (aged 18–40 years), greater midfoot mobility has been associated with successful outcomes of foot orthoses use at 6 and 12 weeks [3, 4]. However, advancing age is associated with greater soft tissue stiffness and less ankle and subtalar joint range of motion [5], with three-dimensional motion analysis demonstrating that older individuals have less foot and ankle mobility during walking compared to younger people [6, 7]. It is plausible that the outcomes and effects of foot orthoses reported in younger adults with greater midfoot mobility and PFP will be different in older populations in the presence of lower foot mobility. Currently, it is unclear whether there are differences in midfoot mobility between younger and older people with PFP.

The aim of this study was to explore differences in midfoot mobility across different age groups of people with PFP. It was hypothesised that older people with PFP would demonstrate less midfoot mobility than younger adults with PFP.

## Methods

### Study design

This cross-sectional analysis utilised baseline participant data from two unique cohorts recruited in Australia. The randomised controlled trial cohort (*n* = 179) was recruited in Brisbane (May 2004 to May 2006), for a study evaluating the effectiveness of foot orthoses for PFP [8]. From this cohort, 110 participants with complete foot mobility datasets were included in the current study. We included an additional 84 participants with PFP from an observational cohort study in Melbourne (July 2012 to March 2015) [9]. Ethical approval was obtained for each study, and all participants provided written informed consent prior to study enrolment.

### Participants

Table 1 details the eligibility criteria for each study used in this analysis. Volunteers were included in either study if they had insidious onset anterior or retro-patellar knee pain, with a severity of at least 30 mm on a 100 mm visual analogue scale (VAS), or pain provoked by at least two activities that load the PF joint (e.g. prolonged sitting or kneeling, squatting, running, hopping, stair ambulation, ambulation or rising from sitting). This is consistent with published recommendations regarding diagnostic criteria for PFP [10]. Volunteers were excluded if they had concomitant injury or pain emanating from the hip, lumbar spine, or other knee structures. Both studies recruited participants via community advertising and referrals from health and medical practitioners.

**Table 1:** Eligibility criteria for the two PFP cohorts

	Collins et al. [8] (*n* = 110)	Observational cohort [9] (*n* = 84)
Inclusion criteria		
Age	18–40 years	18–50 years
Symptoms	Insidious onset anterior knee or retro-patellar pain, aggravated by at least 2 of the following activities: prolonged sitting or kneeling, squatting, running, hopping, stair ambulation	Anterior or retro-patellar knee pain aggravated by at least 2 patellofemoral joint loading activities (e.g. stair ambulation, squatting, rising from sitting) on most days during the past month
	Tenderness on patellar palpation, or pain with step-down/double-leg squat	
Pain severity	Worst pain over the preceding week ≥30 mm on a 100 mm VAS	≥30 mm on a 100 mm VAS during aggravating activities
Duration of PFP	≥ 6 weeks	≥ 3 months
Exclusion criteria		
Other injury or surgery	Concomitant injury/pain from the lumbar spine, hip or other knee structures	Concomitant pain from the lumbar spine, hip or other knee structures
	Previous knee surgery	Planned or previous knee surgery
	Patellofemoral instability	Moderate to severe concomitant TFJ OA (KL grade > 3 on AP radiograph)
	Knee joint effusion	
	Foot conditions precluding foot orthoses use	
Interventions	Physical therapy or foot orthoses (previous 12 months)	Knee injections (previous 3 months)
	Anti-inflammatory drugs	
Other	Strapping tape allergy	Contraindications to x-ray (e.g. pregnancy, breastfeeding)
	Unable to understand written/spoken English	Unable to understand written/spoken English
		Physically unable to undertake testing procedures

*PFP* patellofemoral pain, *VAS* visual analogue scale, *TFJ* tibiofemoral joint, *OA* osteoarthritis, *KL* Kellgren-Lawrence, *AP* anteroposterior

### Characterisation of patellofemoral pain

Both PFP cohorts were characterised using the same reliable and valid measures [2]. Usual and worst knee pain severity over the previous week was measured using two separate 100 mm visual analogue scales (VAS), where 0 mm represented ‘no pain’, and 100 mm represented ‘worst pain imaginable’. Participants were asked to place a vertical mark through the horizontal line that represented their usual/worst pain severity, and the distance was recorded in millimeters. The Anterior Knee Pain Scale (AKPS) comprises 13 items related to limping, weight-bearing, walking, stair ambulation, squatting, running, jumping, prolonged sitting with knees flexed, pain, swelling, painful patellar movements, thigh muscle atrophy, and flexion deficiency [11]. For each item, participants selected the response that best described their knee pain. All items were summated to produce a final score from 0 to 100, where zero represented ‘maximal disability’ and 100 represented ‘no disability’.

### Foot mobility measures

**Fig. 1:**
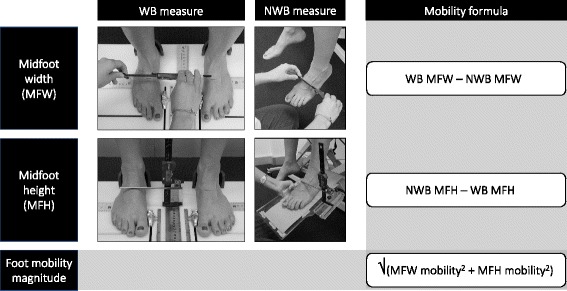
Midfoot mobility measures

A single assessor (NJC) used reliable methods to quantify midfoot mobility, which have been detailed previously [12] (Fig. 1). Briefly, weight-bearing (WB) measures were taken with participants in relaxed standing on a custom-made foot measurement platform [13], with participants asked to maintain equal body weight on each foot while measures were taken (self-monitored). The dorsum of the foot was marked at 50% of the total foot length. Midfoot height and midfoot width were measured at 50% foot length using digital calipers. Non-weight bearing (NWB) measures were then taken with the participant seated on a plinth, with the femur of their test limb horizontal, tibia vertical, and foot and ankle relaxed. A custom-made platform was positioned under the plantar surface of the foot, with minimal contact. The participant was asked to provide feedback to ensure equal contact under the anterior, posterior, medial and lateral aspects of the plantar surface. Midfoot height was measured at 50% foot length using digital callipers fixed to the platform. Non-weight bearing midfoot width was measured at 50% foot length with digital callipers. Weight-bearing and non-weight bearing measures were used to calculate three measures of foot mobility. Midfoot height mobility was calculated as the difference in midfoot height from NWB to WB. Midfoot width mobility was calculated as the difference in midfoot width from WB to NWB. Foot mobility magnitude was then calculated to provide a composite value of midfoot mobility in the vertical and mediolateral directions (√[midfoot height mobility^2^ + midfoot width mobility^2^]).

### Statistical analysis

Cluster analysis (using the K-means algorithm) was used to classify participants into three homogenous groups based on age. This was due to the skewed age distribution across the cohort. Due to the lower proportion of older participants, splitting into tertiles would have produced age categories that may not be homogenous and reflect expected physiological characteristics (for example, 34–50 years). Visual inspection confirmed that the age clusters formed were clinically appropriate (i.e. aligned with decades). After cluster formation, univariate analyses of covariance (ANCOVA) were used to compare midfoot height mobility, midfoot width mobility, and foot mobility magnitude between the three age groups. Sex and weight were entered as covariates in all analyses. Post-hoc tests were conducted where significant main effects were detected, using Bonferroni adjustment for multiple comparisons. Between-group differences in foot mobility were expressed as mean differences with 95% confidence intervals (CIs). Effect sizes (with 95% CIs) were calculated as the between-group difference in means, divided by the pooled standard deviation, and interpreted as small 0.2 to 0.6, moderate 0.6 to 1.2, large 1.2 to 2.0, and very large > 2.0 [14]. Statistical analyses were conducted using SPSS version 24 (IBM Corp, NY, USA), and significance was set at 0.05.

## Results

One hundred ninety four participants with PFP (113 (58%) women, mean ± SD age 32 ± 7 years, height 1.7 ± 0.1 m, weight 74 ± 17 kg, BMI 25 ± 4.9 kg/m^2^) were included (Table 2). The majority of participants had experienced their PFP symptoms for at least two years (≤3 months: 11 participants [6%]; 4–6 months: 11 participants [6%]; 7–12 months: 36 participants [19%]; 1–2 years: 22 participants [11%]; > 2 years: 113 participants [58%]). On average, participants reported usual pain severity of 27 ± 22 mm, worst pain severity of 38 ± 24 mm, and AKPS 72 ± 12 (Table 2).

**Table 2:** Participant characteristics (values are mean (standard deviation) unless otherwise stated)

	18–29 years (*n* = 70)	30–39 years (*n* = 101)	40–50 years (*n* = 23)	Total cohort (*n* = 194)
Age, sex, and anthropometrics				
Number of females (%)	43 (61.4)	58 (57.4)	12 (52.2)	113 (58.2)
Age (years)	24.7 (3.1)	33.9 (2.7)	45.4 (3.5)	31.9 (7.2)
Height (cm)	171.8 (9.4)	172.2 (8.8)	168 (7.4)	171.5 (8.9)
Weight (kg)	73.2 (18.5)	73.5 (15.6)	78.4 (13.8)	74 (16.5)
Body mass index (kg/m^2^)	24.7 (5.3)	24.7 (4.4)	27.8 (5.3)	25 (4.9)
Duration of symptoms [*n* (%)]				
0–3 months	4 (5.8)	7 (6.9)	0 (0)	11 (5.7)
4–6 months	3 (4.3)	5 (5)	3 (13)	11 (5.7)
7–12 months	12 (17.4)	20 (19.8)	4 (17.4)	36 (18.7)
1–2 years	11 (15.9)	10 (9.9)	1 (4.3)	22 (11.4)
> 2 years	39 (56.5)	59 (58.4)	15 (65.2)	113 (58.5)
Participant characteristics				
Usual pain VAS (0–100)	37.2 (19.3)	28.9 (17.2)	26.6 (22.4)	31.6 (19)
Worst pain VAS (0–100)	56.7 (18.9)	49.2 (23.6)	38.2 (23.6)	50.6 (22.6)
Anterior Knee Pain Scale (100–0)	74.1 (9.9)	72.3 (11.3)	72.2 (12.3)	72.9 (10.9)

*VAS* visual analogue scale

Cluster analysis identified three age groups: 18–29 years (*n* = 70); 30–39 years (*n* = 101); and 40–50 years (*n* = 23). Table 3 presents foot mobility measures for each group, along with between-group differences and effect sizes. There was a significant main effect for age for midfoot height mobility (*p* < 0.001) and foot mobility magnitude (*p* = 0.007). Post-hoc tests revealed that midfoot height mobility differed significantly between all three groups (see Table 2), while those 40–50 years had significantly less foot mobility magnitude than those aged 18–29 years (moderate effect size). There were no significant main effects for age for midfoot width mobility (*p* > 0.05).

**Table 3:** Between-group differences and effect sizes in foot mobility (with 95% confidence intervals)

	Foot mobility (mean [SD])			Between-group differences (95% CI)					
	18–29 years (*n* = 70)	30–39 years (*n* = 101)	40–50 years (*n* = 23)	18–29 v 30–39		18–29 v 40–50		30–39 v 40–50	
				MD	SMD	MD	SMD	MD	SMD
Midfoot width difference (mm)	9.4 (3.8)	9.5 (3.4)	9.8 (3.4)	0.1 (−1.2 to 1.3)	−0.03 (−0.33 to 0.28)	0.2 (−1.8 to 2.2)	− 0.11 (− 0.58 to 0.36)	0.2 (− 1.8 to 2.1)	− 0.09 (− 0.54 to 0.36)
Midfoot height difference (mm)	14.8 (3.4)	13.6 (3.0)	11.7 (3.4)	1.3 (0.1 to 2.4)*	0.38 (0.07 to 0.69)*	3 (1.2 to 4.9)*	0.91 (0.42 to 1.4)*	1.8 (0.00 to 3.6)*	0.62 (0.16 to 1.1)*
Foot mobility magnitude (mm)	17.9 (3.9)	16.9 (3.4)	15.5 (4.1)	1.1 (−0.2 to 2.4)	0.27 (−0.03 to 0.58)	2.7 (0.6 to 4.8)*	0.61 (0.13 to 1.09)*	1.6 (− 0.5 to 3.7)	0.4 (− 0.06 to 0.85)

*p < 0.05; positive effect size indicates smaller value in older group

*MD* mean difference, *SMD* standardised mean difference

## Discussion

This study observed that in individuals with PFP, those aged 40–50 years had less foot mobility than younger adults aged 18–29 years, as evidenced by measures of midfoot height mobility and foot mobility magnitude. These differences represented a moderate effect size, and exceed the intra-rater minimal detectable change (MDC 95%) associated with these measures (midfoot height mobility 2 mm; foot mobility magnitude 3.1 mm [12]). The differences between age groups were specific to both midfoot height mobility and foot mobility magnitude; however, there were no differences in midfoot width mobility.

The finding of less foot mobility in the older age groups is consistent with previous studies that have compared various measures of foot posture and function in healthy older versus younger individuals [5, 6, 15]. Menz [5] concluded from his review that ankle dorsiflexion-plantarflexion and subtalar joint inversion-eversion range of motion are 12–30% lower in older individuals, and Lee et al. [15] found that range of motion in the sagittal plane of the forefoot was lower in older compared to younger healthy women. Furthermore, Arnold et al. [6] used three-dimensional motion analysis of a multi-segment foot model to demonstrate that older people exhibited less sagittal plane motion of the midfoot during gait than younger people, which parallels our finding of reduced midfoot height mobility.

It is important to note that our cohort had an upper age limit of 50 years, which is considerably lower than previous age-related foot kinematic studies that observed individuals up to 86 years of age. Beyond 50 years, stiffness of the foot increases due to changes in plantar soft tissues and joint ranges of motion [5]. Therefore, it is possible that there may be greater reductions in foot mobility in those aged 50 years or older with PFP, and that our findings potentially underestimate the amount of foot mobility present in older individuals with PFP.

Less midfoot mobility in older adults with PFP may have both clinical and research implications. Foot orthoses have been shown to be an effective intervention for PFP [8], more so in those with greater midfoot width mobility [3, 4]. Interestingly, we did not find differences in midfoot width mobility, which might indicate that the association between success with orthoses and midfoot mobility is not related to age. Furthermore, as motion control capabilities are not the only means of orthoses effectiveness [16], foot orthoses may be beneficial for older people with PFP displaying less foot mobility due to their ability to redistribute plantar pressures [17, 18] and attenuate plantar loads during weight bearing [5]. This requires further exploration in an older PFP cohort.

Notwithstanding the possible benefits of measuring midfoot mobility, there are several limitations of our study that should be considered in generalising to the clinical context. Firstly, we used a custom-made platform that cannot be purchased commercially. Simple digital callipers can be used to measure midfoot width at 50% foot length in weight bearing and non-weight bearing. However, measurement of midfoot height requires a flat, firm base to be in contact with the sole of the foot during measurement. This necessitates specific equipment, particularly for the non-weight bearing measure. Until such a device is commercially available, clinicians can use the Foot Posture Index to quantify foot posture and mobility. Cornwall and McPoil [19] demonstrated that people classified as more ‘pronated’ on the FPI (higher score) had greater midfoot height mobility, midfoot width mobility and foot mobility magnitude compared to people classified as ‘supinated’ (lower score). Secondly, it is important to note that the foot mobility measures we have used are ‘quasi-dynamic’, in that they document changes in foot posture from relaxed sitting to full weight bearing. Although such measures provide useful insights into how the foot responds to loading and may have some value in estimating foot posture during gait [20], they cannot be considered to be equivalent to true kinematic measures obtained with motion analysis systems. Thirdly, we used data from two existing cohorts, which had a larger proportion of participants aged 30–39 years than 40–50 years or 18–29 years. Despite using two cohorts with a disparity in age range, this may in fact provide us with a more representative age demographic of individuals who suffer from PFP. Finally, our age range of 18–50 years means that we are unable to make generalisations regarding foot mobility to older or younger individuals with PFP. Our findings provide preliminary data regarding the importance of further exploring age-related differences in persons with PFP across the entire lifespan, including adolescents younger than 18 years of age and adults aged over 50 years, to gain a better understanding of age-related differences in foot mobility, and how this may influence treatment response.

## Conclusion

Individuals with PFP aged 40–50 years exhibit less midfoot mobility (based on midfoot height and foot mobility magnitude) than younger adults (18–29 years) with PFP. These differences in midfoot mobility should be considered when planning physical treatment using foot orthoses in individuals with PFP, given there were no differences in midfoot width mobility. Further investigation of midfoot mobility in people of all ages with PFP is warranted.

## Abbreviations

AKPS: Anterior knee pain scale
ANCOVA: Analyses of covariance
CI: Confidence interval
MDC: Minimal detectable change
NWB: Non-weight bearing
PFP: Patellofemoral pain
VAS: Visual analogue scale
WB: Weight-bearing
